# Activation of Voltage-Gated Na^+^ Current by GV-58, a Known Activator of Ca_V_ Channels

**DOI:** 10.3390/biomedicines10030721

**Published:** 2022-03-20

**Authors:** Hsin-Yen Cho, Pei-Chun Chen, Tzu-Hsien Chuang, Meng-Cheng Yu, Sheng-Nan Wu

**Affiliations:** 1 Department of Physiology, National Cheng Kung University Medical College, Tainan City 70101, Taiwan; s36094083@gs.ncku.edu.tw (H.-Y.C.); pcchen@mail.ncku.edu.tw (P.-C.C.); s36091051@gs.ncku.edu.tw (T.-H.C.); s36101042@gs.ncku.edu.tw (M.-C.Y.); 2 Institute of Basic Medical Sciences, National Cheng Kung University Medical College, Tainan City 70101, Taiwan

**Keywords:** GV-58 ((2R)-2-[(6-{[(5-methylthiophen-2-yl)methyl]amino}-9-propyl-9H-purin-2-yl)amino]butan-1-ol), voltage-gated Na^+^ current, resurgent Na^+^ current, window Na^+^ current, K^+^ current, current kinetics, pituitary cell, motor neuron-like cell

## Abstract

GV-58 ((2*R*)-2-[(6-{[(5-methylthiophen-2-yl)methyl]amino}-9-propyl-9H-purin-2-yl)amino]butan-1-ol) is recognized to be an activator of N- and P/Q-type Ca^2+^ currents. However, its modulatory actions on other types of ionic currents in electrically excitable cells remain largely unanswered. This study was undertaken to explore the possible modifications caused by GV-58 in ionic currents (e.g., voltage-gated Na^+^ current [*I*_Na_], A-type K^+^ current [*I*_K(A)_], and *erg*-mediated K^+^ current [*I*_K(erg)_]) identified from pituitary GH_3_ lactotrophs. GH_3_ cell exposure to GV-58 enhanced the transient and late components of *I*_Na_ with varying potencies; consequently, the EC_50_ values of GV-58 required for its differential increase in peak and late *I*_Na_ in GH_3_ cells were estimated to be 8.9 and 2.6 μM, respectively. The *I*_Na_ in response to brief depolarizing pulse was respectively stimulated or suppressed by GV-58 or tetrodotoxin, but it failed to be altered by ω-conotoxin MVIID. Cell exposure to this compound increased the recovery of *I*_Na_ inactivation evoked by two-pulse protocol based on a geometrics progression; however, in its presence, there was a slowing in the inactivation rate of current decay evoked by a train of depolarizing pulses. The existence of GV-58 also resulted in an increase in the amplitude of ramp-induced resurgent and window *I*_Na_. The presence of this compound inhibited *I*_K(A)_ magnitude, accompanied by a shortening in inactivation time course of the current; however, it mildly decreased *I*_K(erg)_. Under current-clamp conditions, GV-58 increased the frequency of spontaneous action potentials in GH_3_ cells. Moreover, in NSC-34 motor neuron-like cells, the presence of GV-58 not only raised *I*_Na_ amplitude but also reduced current inactivation. Taken together, the overall work provides a noticeable yet unidentified finding which implies that, in addition to its agonistic effect on Ca^2+^ currents, GV-58 may concertedly modify the amplitude and gating kinetics of *I*_Na_ in electrically excitable cells, hence modifiying functional activities in these cells.

## 1. Introduction

GV-58 ((2*R*)-2-[(6-{[(5-methylthiophen-2-yl)methyl]amino}-9-propyl-9H-purin-2-yl)amino]butan-1-ol), which was developed as a modification of (R)-roscovitine, has been viewed as an opener of N- and P/Q-type Ca^2+^ channels [1,2,3,4,5]. This compound was presumably thought to slow the closing of the voltage-gated Ca^2+^ (Ca_V_) channel, resulting in a large increase in total Ca^2+^ entry during motor nerve action potential activity [1]. Its presence was reported to enhance spontaneous and evoked activity from murine ventral horn cultures on microelectrode arrays [4]. Earlier studies have demonstrated that this compound is effective for the management of neuromuscular weakness, such as Lambert–Eaton myasthenic syndrome [3,6,7,8,9]. However, the issue of whether and how other types of ionic currents could be perturbed by this compound remains largely unknown.

It has been established that nine isoforms (i.e., Na_V_1.1–1.9 [or SCN1A-SCN5A and SCN8A-SCN11A]) of voltage-gated Na^+^ (Na_V_) channels are expressed in mammalian excitable tissues, which include central or peripheral nervous system and endocrine system [10,11]. The imperative role played by the activity of these channels is to depolarize the membrane and generate the upstroke of the action potential (AP), hence controlling the firing amplitude, frequency, and pattern present in excitable cells [10,11,12]. Of additional interest, several activators of Na_V_ channels have been elaborated preferentially to slow the inactivation rate as well as to increase the late component of voltage-gated Na^+^ current (*I*_Na_), such as tefluthrin, telmisartan, and apocynin [13,14,15], whereas some Na_V_ channel inhibitors (e.g., ranolazine and esaxerenone) known to increase the inactivation rate of the current have been demonstrated [16,17]. However, at present, whether the existence of GV-58 is capable of interacting with the Na_V_ channels to modify the magnitude and gating properties of *I*_Na_ in excitable cells remains not very thoroughly studied, although its effectiveness in stimulating N- and P/Q-type Ca^2+^ currents has been demonstrated [1,2,3,4,5].

In light of the above-mentioned considerations, in this study, the electrophysiological effects of GV-58 and other related compounds in pituitary GH_3_ cells were extensively investigated. We sought to (a) evaluate whether the presence of GV-58 causes any modifications in the amplitude and gating of *I*_Na_ intrinsically in GH_3_ cells; (b) compare the effect of this compound on the peak and late components of *I*_Na_ activated in response to brief depolarizing pulse; (c) study the effect of GV-58 on *erg*-mediated K^+^ current (*I*_K(erg)_); (d) assess its possible perturbations on spontaneous APs; and (e) investigate the effect of GV-58 on *I*_Na_ in NSC-34 motor neuron-like cells. Our results provide the evidence to reveal that, notwithstanding its ability to stimulate the activity of voltage-gated Ca^2+^ (Ca_V_) channels, the differential stimulation by GV-58 of peak and late *I*_Na_ may participate in its regulation of the electrical behaviors of excitable cells (e.g., GH_3_ and NSC-34 cells).

## 2. Materials and Methods

### 2.1. Chemicals, Drugs, and Solutions

GV-58 (1402821-41-3, CHEMBL2206242, SCHEMBL17628602, (2*R*)-2-[(6-{[(5-methylthiophen-2-yl)methyl]amino}-9-propyl-9H-purin-2-yl)amino]butan-1-ol, C_18_H_26_N_6_OS, https://pubchem.ncbi.nlm.nih.gov/compound/71463101, accessed on 1 December 2022), ω-conotoxin MVIID, and tetrodotoxin (TTX) were acquired from Alomone Labs (Genechain, Kaohsiung, Taiwan), while 4-aminopyridine, E-4031, nimodipine, ranolazine, retinoic acid, riluzole, and tetraethylammonium chloride (TEA) were from Sigma-Aldrich (Merck, Taipei, Taiwan). The stock solution of GV-58 was kept under −20 °C for long-term storage. We obtained culture media (e.g., Ham’s F-12 medium), fetal bovine or calf serum, horse serum, L-glutamine, and trypsin/EDTA from Hyclone^TM^ (Thermo Fisher Scientific, Tainan, Taiwan), and all other chemicals, including CsCl, CsOH, CdCl_2_, and EGTA, were of laboratory grade and supplied from standard sources.

The external solutions and pipette solutions used in this work are illustrated in Table 1.

### 2.2. Cell Preparations

Pituitary GH_3_ somatolactotrophs, supplied by the Bioresource Collection and Research Center ([BCRC-60015, https://catalog.bcrc.firdi.org.tw/BcrcContent?bid=60015, accessed on 1 December 2022], Hsinchu, Taiwan), were maintained in Ham’s F-12 medium, with which 15% heat-inactivated horse serum (*v/v*), 2.5% fetal calf serum (*v/v*), and 2 mM L-glutamine were supplemented. NSC-34 neuronal cells were originally produced by fusion of motor neuron-enriched, embryonic mouse spinal cords with mouse neuroblastoma [12,18]. They underwent growth in a 1:1 mixture of DMEM and Ham’s F12 medium with 10% heat-inactivated fetal bovine serum (*v/v*) and 1% penicillin-streptomycin. To slow cell proliferation and enhance their maturation towards a differentiated state, before confluence, we maintained NSC-34 cells in 1:1 DMEM plus Ham’s F-12 medium supplemented with 1% fetal bovine serum and 1 μM retinoic acid. GH_3_ and NSC-34 cells were grown in monolayer culture in 50 mL plastic culture flasks in a humidified environment of CO_2_/air (1:19). Subcultures were made by trypsinization (0.025% trypsin solution [HyClone^TM^] containing 0.01% sodium *N*,*N*,-diethyldithiocarbamate and EDTA). We conducted the electrophysiological measurements when cells reached 60–79% confluence (usually 5–7 days).

### 2.3. Electrophysiological Measurements

During the few hours before the experiments, we gently dissociated GH_3_ or NSC-34 cells with 1% trypsin-EDTA solution and then placed a few drops of cell suspension into a home-made chamber fixed on the working stage of an inverted DM-II fluorescence microscope (Leica; Major Instruments, Tainan, Taiwan). Cells were immersed at room temperature (20–25 °C) in normal Tyrode’s solution, the composition of which was detailed in Table 1, and they were allowed to attach to the chamber’s bottom. The electrodes that we used to measure were fabricated from Kimax-51 glass tubing with 1.5 mm outer diameter (#34500; Kimble, Dogger, New Taipei City, Taiwan) by using a vertical two-stage puller (PP-830; Narishige, Major Instruments, Tainan, Taiwan). Being filled with different internal solution, the recording electrodes had tip resistances of 3–5 MΩ. During the measurements, the electrode was mounted in an air-tight holder which has a suction port on the side, and a chloride silver was used to be in contact with the internal solution. We recorded ionic currents or membrane potential in the whole-cell configuration of a modified patch-clamp technique with the use of either an Axoclamp-2B (Molecular Devices, Sunnyvale, CA, USA) or an RK-400 amplifier (Bio-Logic, Claix, France), as elaborated elsewhere [13,14,15]. GΩ-seals were commonly achieved in an all-or-nothing fashion and resulted in a dramatic improvement in signal-to-noise ratio. The liquid junction potentials (−13 ± 1 mV, n = 27), which occur when the ionic compositions in the pipette internal solution and those of bath solution are different, were zeroed shortly before GΩ-seal formation was achieved, and the whole-cell data were then corrected. During measurements, we exchanged the solutions through a home-made gravity-driven type of bath perfusion.

The signals (i.e., current and potential tracings) were monitored at a given interval and digitally stored on-line at 10 kHz in an ASUS ExpertBook laptop computer (P2451F; Yuan-Dai, Tainan, Taiwan). For efficient analog-to-digital (A/D) and digital-to-analog (D/A) conversion to proceed, a Digidata-1440A equipped with a laptop computer was operated by pClamp 10.6 software run under Microsoft Windows 7 (Redmond, WA, USA). We low-pass filtered current signals at 2 kHz with a FL-4 four-pole Bessel filter (Dagan, Minneapolis, MN, USA). A variety of pClamp-generated voltage-clamp protocols with various rectangular or ramp waveforms were designed and then delivered to the tested cells through D/A conversion in order to determine the current–voltage (*I–V*) relation of ionic currents specified (Section 3.3 and Section 3.10) and the recovery time course of current inactivation (Section 3.4). As pulse-train stimulation was applied to the tested cell, we used an Astro-Med Grass S88X dual output pulse stimulator (Grass, West Warwick, RI, USA).

### 2.4. Data Analyses

To assess concentration-dependent stimulation of GV-58 on the transient (peak) and late components of *I*_Na_, we kept cells bathed in Ca^2+^-free Tyrode’s solution. During the recordings, we voltage-clamped each examined cell at a holding potential of −80 mV, and the brief depolarizing pulse to −10 mV was delivered to evoke peak and late *I*_Na_. The late *I*_Na_ during cell exposure to 100 μM GV-58 was taken as 100%, and those (i.e., peak and late *I*_Na_) acquired in the presence of different GV-58 concentrations (0.3–30 μM) were then compared. The concentration-dependent stimulation by this compound of peak and late *I*_Na_ in pituitary GH_3_ cells were determined by fitting the modified Hill equation. That is,

$$Percentage\;increase\;\left(\%\right)=\left(E_{max}\times {\left[GV-58\right]}^{n_{H}}\right)/\left(EC_{50}^{n_{H}}+{\left[GV-58\right]}^{n_{H}}\right)$$

where [*GV*-58] is the *GV*-58 concentration applied, *n_H_* the Hill coefficient, *EC*_50_ the concentration required for a 50% stimulation of peak or late *I*_Na_ activated in response to rapid depolarizing command voltage, and *E*_max_ the maximal stimulation of peak or late *I*_Na_ produced by the existence of *GV*-58.

The *I–V* relationship of peak *I*_Na_ with or without addition of *GV*-58 was derived and fitted with a Boltzmann function given by:

$$\frac{I}{I_{max}}=\frac{G}{1+exp\left[-\left(V-V_{h}\right)/k\right]}\times \left(V-E_{rev}\right)$$

where *V* is the voltage in mV, *E_rev_* the reversal potential of *I*_Na_ (fixed at +45 mV), *G* the Na^+^ conductance in nS, and *I* the current in pA, while *V_h_* and *k* are the gating parameters.

### 2.5. Curve-Fitting Procedures and Statistical Analyses

Curve-fitting (linear or non-linear [e.g., exponential or sigmoidal curve]) to experimental datasets was conducted with the goodness of fit by using different analytical procedures, such as the Microsoft “Solver” built in Excel 2019 (Microsoft), OriginPro^®^ 2021 program (OriginLab; Scientific Formosa, Kaohsiung, Taiwan), and MathCad Prime 7 (Otsuka, New Taipei City, Taiwan). The electrophysiological data are presented as the mean ± standard error of the mean (SEM), with the sample sizes (n) indicating the cell numbers from which the results were acquired. We performed Student’s *t*-tests (for paired or unpaired samples) and analysis of variance (one-way ANOVA or two-way-ANOVA) with or without repeated measures followed by post hoc Fisher’s least significance difference test. The statistical calculations were performed by using SPSS 17.0 (AsiaAnalytics, Taipei, Taiwan). A *p*-value of less than 0.05 was considered to indicate statistical difference.

## 3. Results

### 3.1. Stimulatory Effect of GV-58 on Voltage-Gated Na^+^ Current Measured from Pituitary GH_3_ Cells

**Figure 1:**
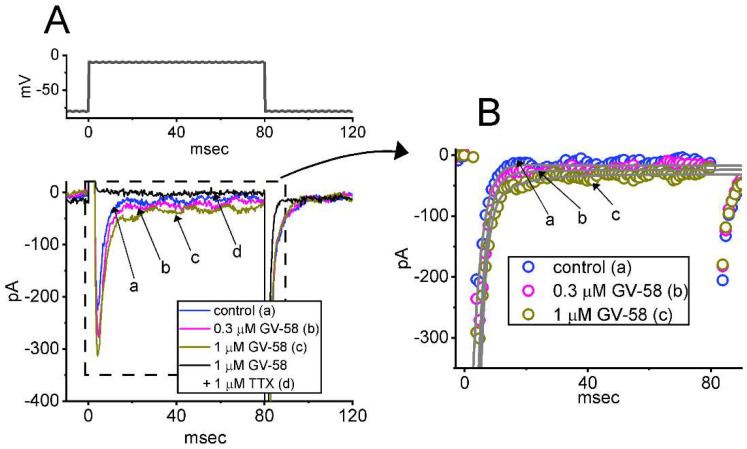

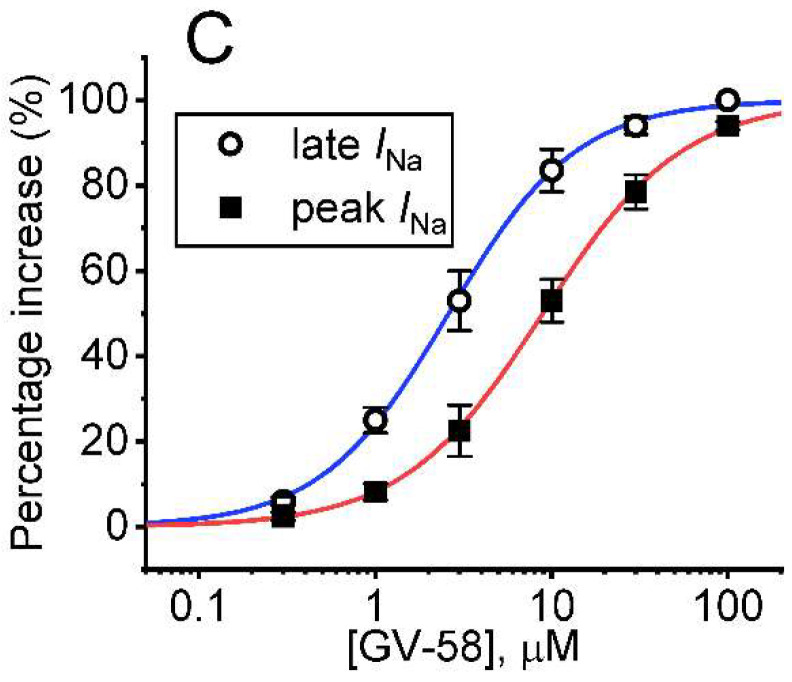
Stimulatory effect of GV−58 on voltage-gated Na^+^ current (*I*_Na_) in pituitary GH_3_ cells. This stage of experiments was conducted in cells which were bathed in Ca^2+^−free Tyrode’s solution, and the solution contained 10 mM tetraethylammonium chloride (TEA) and 0.5 mM CdCl_2_. The recording electrodes that we used were filled up with a Cs^+^−enriched solution. (**A**) Representative current traces activated by short depolarizing pulse (indicated in the upper part). Current trace labeled “a” was obtained during control (i.e., absence of GV-58), those labeled “b” or “c” were recorded during the exposure to 0.3 or 1 μM GV−58, and that labeled “d” was obtained in the presence of 1 μM GV-58 plus 1 μM tetrodotoxin (TTX). In (**B**), current traces (indicated in open circles) indicate an expanded record from dashed box of (A) for a higher time resolution. Of note, current trace labeled “d” was skipped. The gray lines overlaid on each trace indicate best fit to the data points with a two-exponential function. (**C**) Concentration−dependent response of GV−58−mediated stimulation of peak or late *I*_Na_ residing in GH_3_ cells (mean ± SEM; n = 8). The peak and late components of *I*_Na_ activated in response to the depolarizing pulse from −80 to −10 mV were taken at the start and the end−pulse of the depolarizing command voltage with a duration of 80 ms. The continuous line (blue and red colors) indicates the best fit to a modified Hill equation, as revealed in Materials and Methods.

For the first stage of measurements, we kept cells bathed in Ca^2+^-free Tyrode’s solution which contained 10 mM tetraethylammonium chloride (TEA) and 0.5 mM CdCl_2_. TEA and CdCl_2_ were used to block most of the K^+^ and Ca^2+^ currents, respectively. As the depolarizing command voltage from −80 to −10 mV was imposed on the tested cells, we were able to detect an inward current which displayed the rapidly activating and inactivating time course (Figure 1A,B). This type of inward current was, respectively, sensitive to inhibition or stimulation by tetrodotoxin (1 μM) or tefluthrin (10 μM), and it has hence been identified as a voltage-gated Na^+^ current (*I*_Na_) [13,17,19]. Of notice, one minute after cells were exposed to GV-58, the amplitude of *I*_Na_ progressively increased in combination with a slowing in the inactivation time course of the current. For example, the addition of GV-58 markedly increased the peak *I*_Na_ from 215 ± 21 to 309 ± 29 pA (n = 8, *p* < 0.05), and the time constant in the slow component of current inactivation (τ_inact(S)_) was concurrently raised to 3.8 ± 0.4 ms from a control of 2.1 ± 0.3 ms (n = 8, *p* < 0.05). However, no significant difference in the fast component of current inactivation could be demonstrated. After washout of the compound, current amplitude was returned to 219 ± 23 pA (n = 7, *p* < 0.05).

The relationship between the GV-58 concentration and the peak or late component of *I*_Na_ evoked in response to abrupt membrane depolarizing was further analyzed and constructed. In this stage of experiments, each cell was rapidly depolarized from −80 to −10 mV, and peak or late *I*_Na_ measured at varying concentrations (0.3–100 μM) of GV-58 were compared. As can be seen in Figure 1C, GH_3_ cell exposure to GV-58 resulted in a concentration-dependent rise in the amplitude of peak and late *I*_Na_. The EC_50_ value for GV-58-stimulated peak or late *I*_Na_ was computed to be 8.9 or 2.6 μM, respectively, and GV-58 at a concentration of 100 μM almost fully increased *I*_Na_. The present results, therefore, reflect that GV-58 is capable of exercising a stimulatory action on depolarization-activated *I*_Na_ in GH_3_ cells and that the late component of *I*_Na_ increases to a greater extent than the peak component of the current in its presence.

### 3.2. Comparisons of Effects of GV-58, ω-Conotoxin MVIID, and Tetrodotoxin (TTX) on Peak I_Na_

**Figure 2:**
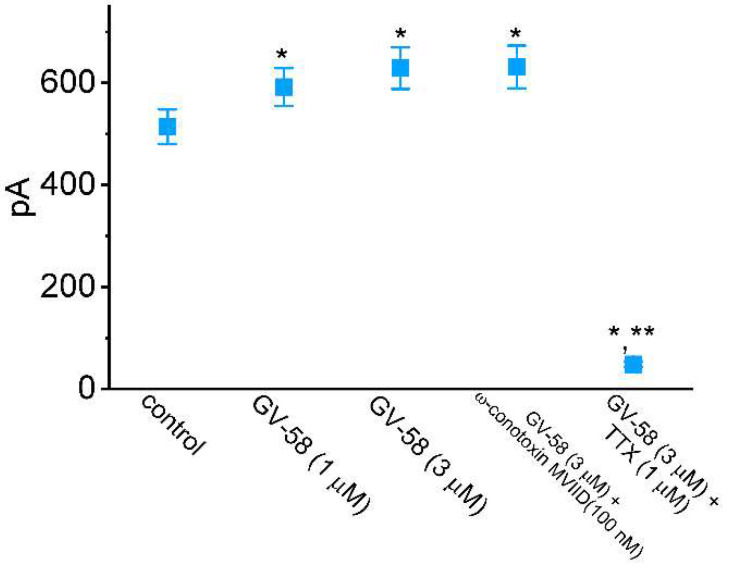
Comparisons of effects of GV-58, GV-58 plus ω-conotoxin MVIID, and GV-58 plus tetrodotoxin (TTX) on the peak amplitude of *I*_Na_ recorded from GH_3_ cells. Cells were immersed in Ca^2+^-free Tyrode’s solution containing 10 mM TEA, and the pipettes used were filled with Cs^+^-containing solution. Current amplitude was taken at the beginning of the brief depolarizing pulse from −80 to −10 mV. Each bar indicates the mean ± SEM (n = 8 for each bar). Of notice, the peak *I*_Na_ in GH_3_ cells is subject to inhibition by TTX but not by ω-conotoxin MVIID. * Significantly different from control (*p* < 0.05) and ** Significantly different from GV-58 (3 μM) alone group (*p* < 0.05). Statistical analyses were made by one-way ANOVA among different groups (*p* < 0.05).

Earlier studies have been demonstrated to report the effectiveness of GV-58 in enhancing the amplitude of voltage-gated Ca^2+^ currents (e.g., N- and P/Q-type Ca^2+^ currents) [2,3,4,5]. We, therefore, explored and compared effects of GV-58, ω-conotoxin MVIID, or TTX on *I*_Na_ inherently in these cells. ω-Conotoxin MVIID, a small, disulfide-rich peptide purified from the venoms of predatory cone snails, was previously reported to be an inhibitor of N- and P/Q-type Ca^2+^ currents in adrenal chromaffin cells [20]. As summarized in Figure 2, the peak amplitude of *I*_Na_ was increased in the presence of GV-58; however, it was effectively depressed by cell exposure to TTX (1 μM) but not by ω-conotoxin MVIID (100 nM). Moreover, the amplitude of L-type Ca^2+^ currents (*I*_Ca,L_) evoked from −50 to +10 mV was not affected by ω-conotoxin MVIID (100 nM) or by GV-58 (3 μM). In the continued presence of 3 μM GV-58, further addition of nimodipine (1 μM), an inhibitor of *I*_Ca,L_, effectively decreased the peak amplitude of *I*_Ca,L_ by about 90%. Therefore, the *I*_Na_ evoked in response to brief depolarizing pulse is sensitive to stimulation by GV-58 and to inhibition by TTX, but it remains unaltered in the presence of ω-conotoxin MVIID, suggesting that the magnitude of N- and P/Q-type Ca^2+^ currents is unlikely to be linked to GV-58-stimulated *I*_Na_ found in GH_3_ cells.

### 3.3. Effect of GV-58 on the Steady-State Current–Voltage (I–V) Relationship of Peak I_Na_

**Figure 3:**
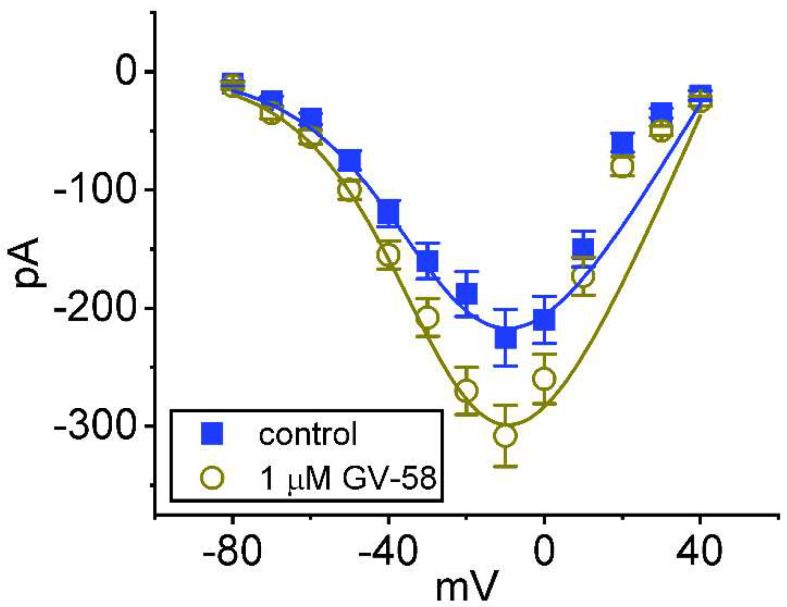
Effect of GV-58 on the steady−state *I–V* relationship of peak *I*_Na_. In these experiments, we voltage−clamped the cells at −80 mV, and various depolarizing pulses from −80 to +40 in 10 mV increments were applied to evoke *I*_Na_. Current amplitude at each depolarizing pulse was measured at the beginning of the voltage pulse. Data points shown in filled blue squares are control, and those in open brown circles were obtained in the presence of 1 μM GV−58. The smooth line taken with or without the GV−58 addition was fitted with a Boltzmann function as detailed in Materials and Methods.

We further examined the steady-state *I–V* relationships of peak I_Na_ obtained with or without the application of GV-58. These voltage-clamp experiments were conducted in cells held at −80 mV, and a series of voltage pulses between −80 and +40 mV was imposed on the tested cells. As shown in Figure 3, the presence of 1 μM GV-58 did not alter the overall *I–V* relationship of peak *I*_Na_, although it increased peak amplitude of *I*_Na_, particularly at the level of −10 mV. The reversal potential of peak *I*_Na_ did not differ significantly between the absence and presence of GV-58. The *I–V* curves obtained in the control period and during exposure to 1 μM GV-58 were fitted with a Boltzmann function as stated in Materials and Methods. In control (i.e., the absence of GV-58), G = 5.5 ± 0.3 nS, V_h_ = −23.9 ± 2.1 mV, k = 14.9 ± 1.9 (n = 8), while in the presence of 1 μM GV-58, G = 7.5 ± 0.4 nS, V_h_ = −23.6 ± 1.9 mV, k = 14.5 ± 1.8 (n = 8). It is, thus, likely that the steady-state activation curve of *I*_Na_ was not changed during cell exposure to 1 μM GV-58.

### 3.4. Effect of GV-58 on the Recovery from I_Na_ Inactivation Evoked during Varying Interpulse Intervals

**Figure 4:**
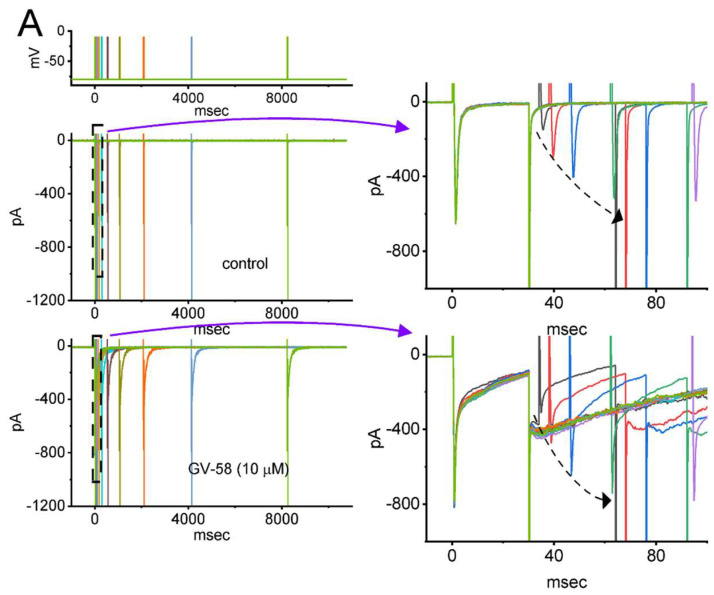

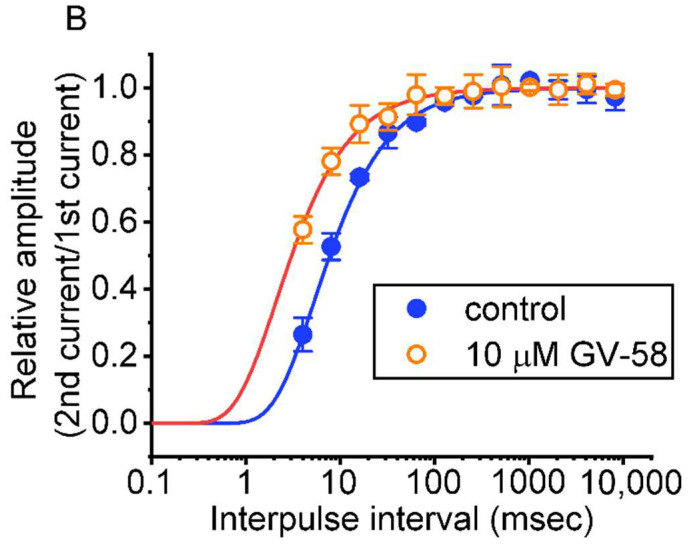
Effect of GV−58 on the recovery of *I*_Na_ inactivation identified in GH_3_ cells. In this set of experiments, as whole−cell configuration was securely established, we applied two−step voltage protocol in a geometrics progression as indicated in the uppermost part of (**A**). (**A**) Representative current traces obtained in the absence (upper) and presence of 10 μM GV−58. The panels on the right side, included to show better illustrations, indicate the expanded records from dashed boxes on the left side. The dashed arrow indicates an incremental progression in the trajectory of current inactivation with the increasing interpulse interval. (**B**) The relationship of interpulse interval versus the relative amplitude taken in the absence (filled blue circles) and presence (open red circles) of 10 μM GV-58 (mean ± SEM; n = 8 for each point). The relative amplitude in the *y*-axis was obtained by dividing the second amplitude activated by 30 ms depolarizing command voltage from −80 to −10 mV by the first one. Of note, the x-axis is illustrated in a logarithmic scale. The smooth curve in the absence and presence of 10 μM GV−58 was fitted to a single-exponential function with time constants of 5.34 and 2.11 ms, respectively. The presence of GV−58 was noted to result in an evident increase in current recovery (i.e., a reduction of recovery time constant).

We next examined whether the existence of GV-58 produces any effect on the recovery of *I*_Na_ from inactivation by responding to a two-step voltage protocol. In this protocol, a 30 ms step to −10 mV was applied to the examined cell, and another 30 ms conditioning step to −10 mV with varying interpulse intervals inactivated most of the current. The recovery from current inactivation at the holding potential of −80 mV was hence examined at different times with a geometrics progression, as demonstrated in Figure 4A,B. In the control period (i.e., GV-58 was not present), the peak amplitude of *I*_Na_ nearly completely recovered from inactivation when the interpulse duration reached 1 s. The time constants of recovery from current inactivation acquired in the absence and presence of 10 μM GV-58 were least squares fitted by a single-exponential function with the values of 5.34 ± 0.07 and 2.11 ± 0.06 ms (n = 8, *p* < 0.05), respectively. The experimental observations indicate that a conceivable increase in the recovery from inactivation of *I*_Na_ is seen by adding GV-58.

### 3.5. GV-58 Effect on I_Na_ Decay Evoked during a Train of Depolarizing Command Voltages in GH_3_ Cells

**Figure 5:**
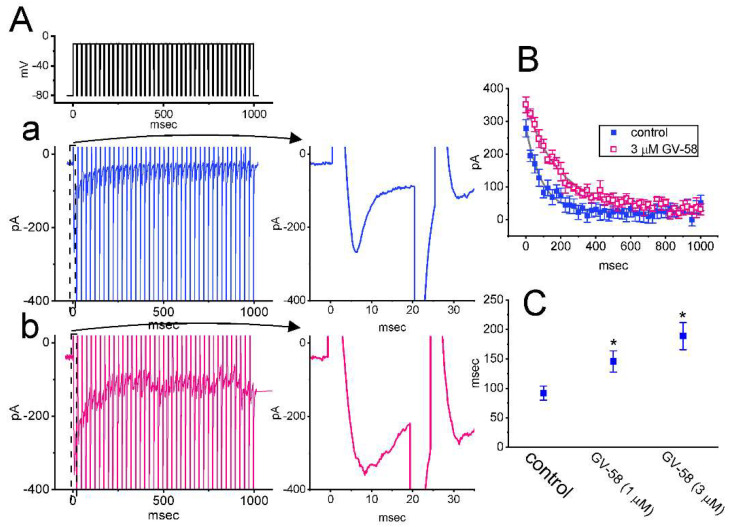
Effect of GV−58 on current decline induced during a 40 Hz train of depolarizing pulses. The train was designed to consist of 40 20 ms pulses separated by 5 ms intervals at −80 mV with a duration of 1 s. (**A**) Representative current traces obtained in the control period (a) and during cell exposure to 3 μM GV−58 (b). The voltage−clamp protocol is illustrated in the uppermost part. To provide single *I*_Na_ trace, the right side of (A) denotes the expanded records from the dashed box of (**Aa**) and (**Ab**). (**B**) The relationship of peak *I*_Na_ versus the train duration in the absence (filled blue symbols) and presence (filled red symbols) of 3 μM GV−58 (mean ± SEM; n = 7 for each point). The continuous smooth lines over which the data points are overlaid are well fitted by single exponential. Of note, the presence of GV−58 can slow the time course of current inactivation in response to a train of depolarizing pulses. (**C**) Summary graph showing effect of GV−58 (1 and 3 μM) on the time constant of current decay in response to a train of depolarizing command voltage from −80 to −10 mV (mean ± SEM; n = 7 for each bar). Current amplitude was measured at the beginning of each depolarizing pulse. Of notice, the presence of GV-58 produces an increase in the time constant in the decline of peak *I*_Na_ activated by a train of pulses. * Significantly different from control (*p* < 0.05).

Previous work has shown the ability of the train of depolarizing pulses to perturb the peak and persistent *I*_Na_, which tend to decrease over time in an exponential fashion [21]. We thus made an effort to explore whether GV-58-mediated stimulation of *I*_Na_ can be perturbed during pulse-train stimulation. In this set of whole-cell current measurements, we delivered a 40 Hz train of depolarizing pulses from −80 to −10 mV to the tested cell. As shown in Figure 5A–C, the slow current inactivation evoked by responding to such pulse-train stimulation was prominently observed. The mean relationship of peak *I*_Na_ versus the train duration taken with or without the addition of 3 μM GV-58 was constructed and then analyzed. It is noticed that the presence of GV-58 results in slowing the time course of current inactivation during such a train of depolarizing pulses. For example, the time constant for *I*_Na_ during a train of command voltages was measurably increased to 189 ± 23 ms (n = 7, *p* < 0.05) from a control value of 92 ± 12 ms (n = 7). It is clear, therefore, that the existence of GV-58 produces a marked increase in the decaying time constant of *I*_Na_ during a train of depolarizing pulses.

### 3.6. Use-Dependence of GV-58-Induced Stimulation of Peak I_Na_

**Figure 6:**
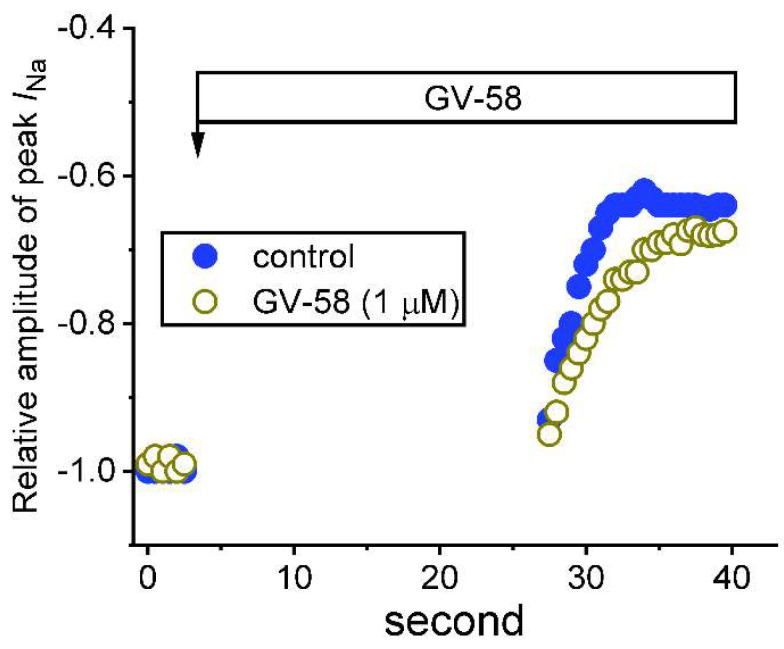
Tonic and use−dependent stimulation of peak *I*_Na_ by GV−58. The cell was held at −80 mV, and the depolarizing pulse from −80 to −10 mV (30 ms in duration) was applied at 2 Hz. The application of GV−58 (1 μM) is illustrated by a horizontal bar shown above. Changes in the relative amplitude of peak *I*_Na_ with or without the addition of GV−58 (1 μM) are illustrated. The peak *I*_Na_ in the absence (filled circles) or presence (open circles) of GV−58 measured during regular repetitive steps at 0.5 Hz was taken as −1.0. Immediately after the voltage pulses were stopped, GV−58 (1 μM) was added to the bath. The repetitive depolarizing pulses to −10 mV at 2 Hz were applied again 25 s after the cessation of command pulses but still in the continued presence of GV−58 (1 μM) (open circles). Of notice, in the presence of GV-58, the peak *I*_Na_ activated by the first depolarizing step following a pause (around 25 s) had been suppressed to a lesser extent (i.e., tonic stimulation), and, during the repetitive stimuli, the amplitude of peak *I*_Na_ was further reduced exponentially to a lesser and slower extent (use−dependent stimulation).

This series of whole-cell experiments was conducted to assess the use-dependent property of GV-58-mediated stimulation of peak *I*_Na_. As shown in Figure 6, the depolarizing pulses from −80 to −10 mV (30 ms in duration) were applied at 0.2 Hz. Under control conditions (i.e., absence of GV-58), when peak *I*_Na_ remained active in Ca^2+^-free Tyrode’s solution, the depolarizing pulses were stopped; thereafter, after 25 s of cessation, the depolarizing pulses from −80 to −10 mV at 2 Hz were given to the cells again. The relative amplitude of peak *I*_Na_ with respect to before the cessation of the depolarizing pulse was constructed and noticed to decline in an exponential fashion. It is illustrated in Figure 5 that peak *I*_Na_ consisted of both tonic and use-dependent components. For example, in the control period, after a 25 s pause, the peak *I*_Na_ evoked by the first voltage step or during subsequent repetitive stimuli was suppressed by 11 ± 3% or 38 ± 5% (n = 7). As cells were exposed to GV-58 (1 μM), the peak *I*_Na_ amplitude at the first depolarizing pulse or during subsequent repetitive pulses were significantly suppressed by 8 ± 2% or 30 ± 4%, respectively; and the decline in peak *I*_Na_ became slowed. The results indicated that GV-58-stimulated *I*_Na_ tends to be composed of two components, namely, tonic and use-dependent components.

### 3.7. Stimulatory Effect of GV-58 on Resurgent I_Na_ (I_Na(R)_) Seen in GH_3_ Cells

**Figure 7:**
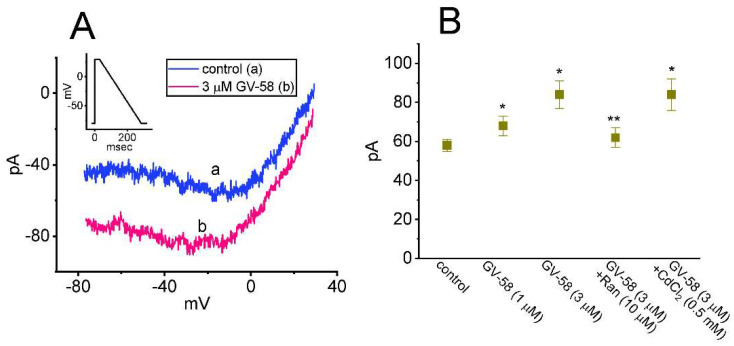
Effect of GV−58 on resurgent *I*_Na_ (*I*_Na(R)_) activated by the downsloping ramp pulse. This set of measurements was conducted in cells bathed in Ca^2+^−free Tyrode’s solution, and we filled up the electrode with Cs^+^−enriched solution. The examined cell was voltage−clamped at −80 mV and the depolarizing pulse to +30 mV for 30 ms; thereafter, a slowly descending ramp from +30 to −80 mV with a duration of 300 ms (i.e., with a ramp slope of −0.37 mV/ms) was applied to evoke *I*_Na(R)._ (**A**) Representative current traces obtained in the control period (a) and during cell exposure to 3 μM GV−58 (b). Inset shows the voltage−clamp protocol delivered. (**B**) Summary graph showing effect of GV−58, GV−58 plus ranolazine (Ran), and GV−58 plus CdCl_2_ on the amplitude of *I*_Na(R)_ (mean ± SEM; n = 8 for each bar). Current amplitudes were measured at the level of −20 mV. Of notice, in the continued presence of GV−58, subsequent application of ranolazine, but not of CdCl_2_, effectively suppresses *I*_Na(R)_ activated by the descending ramp pulse. * Significantly different from control (*p* < 0.05) and ** significantly different from GV−58 (3 μM) alone group (*p* < 0.05). Statistical analyses were performed by one−way ANOVA among different groups (*p* < 0.05).

The *I*_Na(R)_ has been recently identified in GH_3_ cells [19,22]. Consistent with previous observations in neurons or endocrine cells [22,23], the biophysical property of this current is that it remains undetectable until the membrane potential is repolarized below 0 mV. In addition to being activated by negative voltage steps (i.e., repolarizing steps) rather than positive voltage steps, *I*_Na(R)_ was noted to activate and decay more slowly than transient *I*_Na_. The *I*_Na(R)_ is thought to help produce rapid depolarization immediately after an AP and is suited for cells that fire spontaneously at a higher firing rate [23]. For these reasons, we additionally studied whether GV-58 could exert any perturbations on this current. As the whole-cell configuration was securely established, the depolarizing pulse from −80 to +30 mV followed by a 300 ms descending (or repolarizing) ramp command voltage to −80 mV was applied to the examined cell. As depicted in Figure 7A,B, the *I*_Na(R)_ amplitude activated by this voltage-clamp protocol was evidently increased. For example, the presence of 3 μM GV-58 conceivably increased *I*_Na(R)_ at −20 mV from 58 ± 3 to 84 ± 7 pA (n = 7, *p* < 0.05). The further addition of ranolazine (10 μM), but still in the presence of 3 μM GV-58, reduced *I*_Na(R)_ amplitude at the same level to 62 ± 5 pA (n = 7, *p* < 0.05). However, the subsequent application of CdCl_2_ (0.5 mM) failed to exert any effects on GV-58-stimulated *I*_Na(R)_ in GH_3_ cells. Ranolazine was previously demonstrated to be an inhibitor of late *I*_Na_ [16,19].

### 3.8. Effect of GV-58 on the Window Component of I_Na_ (I_Na(W)_) Recorded from GH_3_ Cells

**Figure 8:**
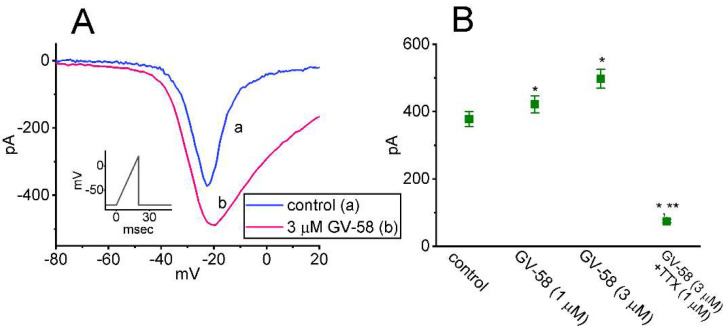
Stimulatory effect of GV−58 on window *I*_Na_ (*I*_Na(W)_) activated in response to the upsloping ramp pulse in GH_3_ cells. In these experiments, the examined cell was held at −80 mV, and we applied the ascending ramp pulse from −80- to +20 mV with a duration of 20 ms (5 mV/ms) to it. (**A**) Representative current traces activated by ramp pulse with a duration of 30 ms during the control period (a) and in the presence of 3 μM GV−58 (b). Inset in (**A**) indicates the voltage−clamp protocol applied. (**B**) Summary graph showing effect of GV−58 and GV−58 plus tetrodotoxin (TTX) on *I*_Na(W)_ amplitude identified in GH_3_ cells (mean ± SEM; n = 8 for each bar). Current amplitudes measured at the level of −20 mV were taken. * Significantly different from control (*p* < 0.05) and ** Significantly different from GV−58 (3 μM) alone group (*p* < 0.05). Statistical analyses were made by one−way ANOVA (*p* < 0.05). Notably, in the continued presence of GV−58, subsequent addition of TTX effectively suppresses the magnitude of *I*_Na(W)_ activated in response to brief ascending ramp pulse.

The presence of instantaneous *I*_Na(W)_ has been previously demonstrated in different types of electrically excitable cells [12,24,25,26]. We continued to explore whether GV-58 presence in GH_3_ cells could modify the magnitude of *I*_Na(W)_ activated in response to rapid ascending ramp command voltage. In an effort to perform these experiments, we voltage-clamped the tested cell at −80 mV, and we then applied an ascending ramp from −80 to +20 mV with a duration of 20 ms to evoke *I*_Na(W)_ [26]. As demonstrated in Figure 8A,B, within one minute of exposing cells to GV-58 (1 or 3 μM), the amplitude of *I*_Na(W)_ achieved by the upsloping ramp with a duration of 20 ms was strikingly increased. For example, the existence of 3 μM GV-58 evidently increased the peak component of *I*_Na(W)_ measured at the level of −20 mV from 378 ± 22 to 498 ± 28 pA (n = 7, *p* < 0.05). After washout of GV-58, current amplitude was returned to 383 ± 24 pA (n = 7). However, it needs to be noticed that *I*_Na(W)_ measured here was evoked by rapid ramp pulse; it would be unlikely to determine the steady-state activation of *I*_Na_. The summary bar graph shown in Figure 8B reveals that the GV-58 addition is effective in increasing the *I*_Na(W)_ amplitude and that subsequent addition of 1 μM TTX suppressed GV-58-mediated increase of *I*_Na(W)_. The present results also imply that GV-58-mediated accentuation of *I*_Na(W)_ is not mediated through its agonistic effect on Ca_V_ channels.

### 3.9. Inhibitory Effect of GV-58 on A-Type K^+^ Current (I_K(A)_) in GH_3_ Cells

**Figure 9:**
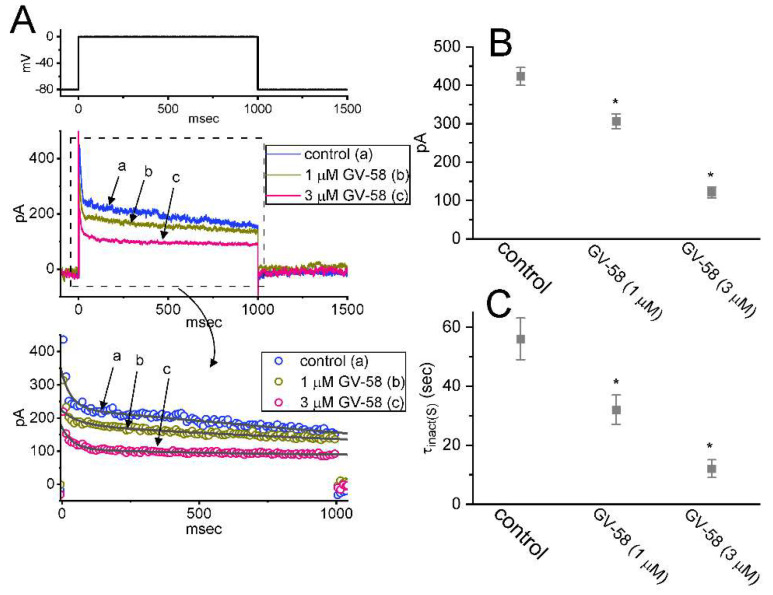
Inhibitory effect of GV−58 on A−type K^+^ current (*I*_K(A)_) in GH_3_ cells. Cells were bathed in Ca^2+^−free Tyrode’s solution containing 1 μM TTX, and we filled up the pipette with K^+^−enriched solution. (**A**) Represent current traces obtained in the control period (“a”, i.e., absence of GV−58) and during cell exposure to 1 μM GV−58 (b) or 3 μM GV-58 (c). The uppermost part shows the voltage-clamp protocol applied. The lower part in (**A**) shows an expanded record from the dashed box, while the data points (circle symbols) were reduced by 50 for better illustration. The smooth line in the lower part represents best fit to two−exponential function (i.e., fast and slow components in current inactivation). Summary graphs appearing in (**B**,**C**) demonstrate inhibitory effects of GV−58 (1 or 3 μM) on peak amplitude and slow component in inactivation time constant (τ_inact(S)_) of *I*_K(A)_, respectively (mean ± SEM; n = 8 for each bar). * Significantly different from controls (*p* < 0.05). Current amplitude was taken at the start of each depolarizing command voltage from −80 to 0 mV with a duration of 1 s. Of notice, cell exposure to GV−58 is capable of decreasing both peak *I*_K(A)_ and the τ_inact(S)_ value of the current in these cells.

The suppression of K^+^ currents was shown to be effective in improving synaptic impairment or muscle weakness in Lambert–Eaton myasthenic syndrome [3,27]. In another separate set of whole-cell current recordings, different types of ionic currents (e.g., *I*_K(A)_) were also further examined with respect to possible modifications of GV-58 on them. In these experiments, we bathed cells in Ca^2+^-free Tyrode’s solution containing 1 μM TTX and 0.5 mM CdCl_2_, while the recording electrode was filled up with K^+^-containing solution. The tested cells were voltage-clamped at −80 mV, and the depolarizing command voltage to 0 mV with a duration of 1 s was delivered to evoke a unique type of K^+^ outward current with rapid activation and inactivation, which is referred to as A-type K^+^ current (*I*_K(A)_) [28]. One minute after cells were exposed to GV-58 (1 or 3 μM), the amplitude of *I*_K(A)_ was found to be conceivably decreased; concurrently, the time constant in the slow component of current inactivation (τ_inact(S)_) was reduced (Figure 9A–C). For example, the addition of 3 μM GV-58 decreased peak *I*_K(A)_ amplitude from 423 ± 23 to 121± 14 pA (n = 8, *p* < 0.05), and it concomitantly reduced the τ_inact(S)_ value from 56 ± 7 to 12 ± 3 ms (n = 8, *p* < 0.05). The presence of 5 mM 4-aminopyridine fully suppressed the amplitude of *I*_K(A)_ in GH_3_ cells. Results from these observations reflect that, like the action of 4-aminopyridine on *I*_K(A)_ described previously [28,29], GV-58 per se is capable of decreasing *I*_K(A)_ and the τ_inact(S)_ value of the current found in GH_3_ cells.

### 3.10. Mild Suppression of Erg-Mediated K^+^ Current (I_K(erg)_) Produced by GV-58

**Figure 10:**
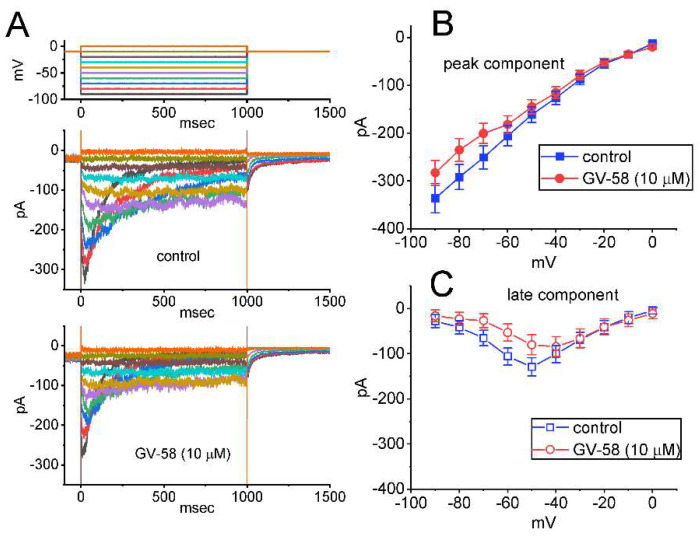
Effects of GV−58 on *erg*−mediated K^+^ current (*I*_K(erg)_) in GH_3_ cells. In these experiments, we kept cells bathed in high-K^+^, Ca^2+^−free solution containing 1 μM TTX, and the patch pipette was filled up with K^+^−containing solution. The composition of these solutions is elaborated in Table 1. (**A**) Representative current traces activated by a series of voltage pulses (indicated in the uppermost part). Current traces shown in the upper part were obtained during the control period, whereas those in the lower part were acquired in the presence of 10 μM GV−58. In (**B**,**C**), the mean current−voltage (*I–V*) relationships of peak (upper, filled symbols) and sustained (lower, open symbols) components of *I*_K(erg)_ achieved in the absence (square symbols) or presence (circle symbols) of 10 μM GV−58 are illustrated, respectively. The peak and sustained components of *I*_K(erg)_ were measured at the beginning and end−pulse of each voltage pulse, respectively. Each bar in (**B**,**C**) represents the mean ± SEM (n = 8). Of notice, the presence of 10 μM GV−58 mildly suppresses the magnitude of *I*_K(erg)_ in these cells.

In another set of experiments, we further examined the effect of GV-58 on *I*_K(erg)_ in GH_3_ cells. This set of measurements was conducted in cells bathed in high-K^+^, Ca^2+^-free solution which contained 1 μM TTX, and the electrode was filled with K^+^-containing solution. The tested cell was maintained in −10 mV, and different command voltages ranging between −90 and 0 mV with a duration of 1 s were applied to evoke hyperpolarization-evoked *I*_K(erg)_ [30,31]. During cell exposure to 3 μM GV-58, the amplitude of *I*_K(erg)_ was noted to remain unaltered. However, as cells were exposed to 10 μM GV-58, the peak and sustained components of *I*_K(erg)_ measured at the entire voltage-clamp ranges examined were diminished (Figure 10). For example, as the hyperpolarizing command voltage from −10 to −90 mV with a duration of 1 s was applied to the tested cell, the peak and sustained components of deactivating *I*_K(erg)_ were significantly reduced to 283 ± 26 and 16 ± 13 pA (n = 8, *p* < 0.05) from control values of 336 ± 31 and 28 ± 14 pA (n = 8), respectively. After washout of the compound, peak and sustained *I*_K(erg)_ was returned to 328 ± 29 and 27 ± 13 pA (n = 7). The mean *I–V* relationships of peak and sustained *I*_K(erg)_ acquired in the absence or presence of 10 μM GV-58 were constructed and are hence presented in Figure 10B,C, respectively. However, as cells were exposed to E-4031 (10 μM), an inhibitor of *I*_K(erg)_, the *I*_K(erg)_ evoked from −10 to −100 mV was fully abolished. Therefore, as compared with its effect on *I*_K(A)_, the *I*_K(erg)_ residing in GH_3_ cells is mildly suppressed by GV-58 presence.

### 3.11. Stimulatory Effect of GV-58 on Spontaneous Action Potentials (Aps) Recorded from GH_3_ Cells

**Figure 11:**
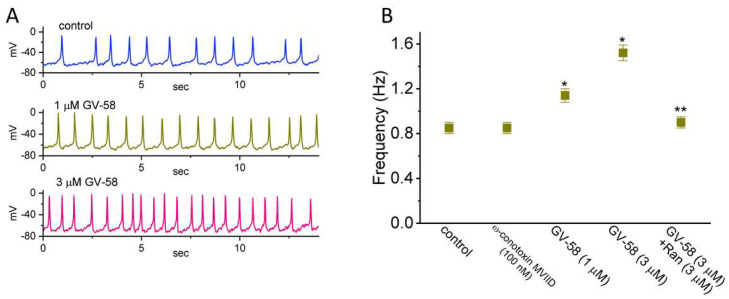
Effects of GV−58 on spontaneous action potentials (APs) recorded from GH_3_ cells. Cells were bathed in normal Tyrode’s solution, and the recording pipettes were filled with K^+^−containing solution. When whole−cell configuration was established, we switched to the whole−cell current clamp recordings to measure changes in membrane potential, as current was set at zero. (**A**) Representative potential traces achieved in the absence (upper) and presence of 1 μM GV−58 (middle) or 3 μM GV−58 (lower). (**B**) Summary graph demonstrating effect of ω−conotoxin MVIID, GV−58, and GV−58 plus ranolazine (Ran) on the firing frequency of APs in GH_3_ cells (mean ± SEM; n = 8). * Significantly different from control (*p* < 0.05) and ** Significantly different from GV−58 (3 μM) alone group (*p* < 0.05). Statistical analyses were made in one−way ANOVA among different groups (*p* < 0.05).

In another set of experiments, we performed current-clamp potential recordings in order to examine any changes in membrane potential of these cells during the absence or presence of GV-58. We kept cells bathed in normal Tyrode’s solution containing 1.8 mM CaCl_2_, and the recording electrode was filled up with K^+^-containing solution. One minute after cells were exposed to GV-58 at a concentration of 1 or 3 μM, the AP firing recorded under current-clamp configuration was progressively raised (Figure 11). For example, GV-58 (3 μM) resulted in a striking increase in the firing frequency of APs from 0.85 ± 0.05 to 1.52 ± 0.07 Hz (n = 7, *p* < 0.05). As the compound was removed, the frequency was returned to 0.88 ± 0.06 Hz (n = 6). However, ω-conotoxin MVIID (100 nM) alone had no effect on AP firing. Moreover, the further addition of ranolazine (10 μM), but in continued presence of 3 μM GV-58, was effective in suppressing the frequency of spontaneous APs observed in GH_3_ cells. It is, thus, possible from the current experiments that GV-58-stimulated increase in AP firing is predominantly linked to its excitatory actions on *I*_Na_, *I*_Na(R)_, and *I*_Na(W)_ yet not solely by the agonistic effect on Ca_V_ channels stated previously [2,3,4,5].

### 3.12. Effect of GV-58 on I_Na_ Inherently in NSC-34 Motor Neuron-like Cells

**Figure 12:**
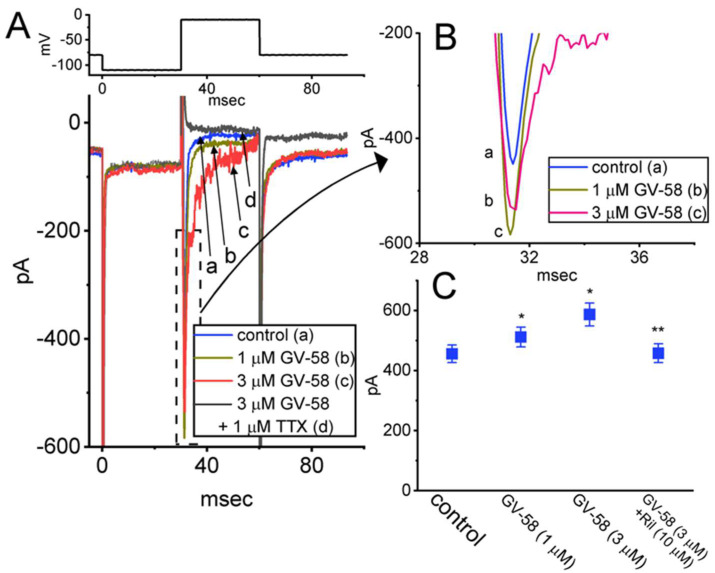
Effects of GV−58 on *I*_Na_ identified in NSC−34 motor neuron−like cells. These experiments were undertaken in cells bathed in Ca^2+^−free Tyrode’s solution which contained 10 mM TEA and 0.5 mM CdCl_2_, while the recording pipettes that we prepared were filled up with Cs^+^−containing solution. (**A**) Representative *I*_Na_ traces obtained in the control (a), during cell exposure to 1 μM GV−58 (b) or 3 μM GV−58 (c), and in the presence of 3 μM GV−58 plus 1 μM TTX (d). The upper part in (**A**) depicts the voltage−clamp protocol applied. In (**B**), an expanded record showing *I*_Na_ traces is taken from the dashed box in (**A**). (**C**) Summary graph showing effect of GV−58 (1 or 3 μM) and GV−58 (3 μM) plus riluzole (Ril, 10 μM). Current amplitude was taken at the beginning of depolarizing command voltage from −100 to −10 mV with a duration of 30 ms. Each bar indicates the mean ± SEM (n = 7). * Significantly different from control (*p* < 0.05) and ** Significantly different from GV−58 (3 μM) alone group (*p* < 0.05). Statistical analyses were made in one−way ANOVA among different groups (*p* < 0.05).

GV-58 was previously reported to enhance spontaneous and evoked activity in the ventral horn of the spinal cord [4]. For these reasons, a final stage of experiments was undertaken to determine whether the *I*_Na_ present in NSC-34 motor neuron-like cells [32] can be vulnerable to any modification by the existence of GV-58. Consistent with the observations made above in GH_3_ cells, the *I*_Na_ in NSC-34 cells can be enhanced during exposure to GV-58 (Figure 12A,B). For example, as NSC-34 cells were exposed to 3 μM GV-58, the peak amplitude of *I*_Na_ activated by abrupt depolarizing pulse from −100 to −10 mV was conceivably raised to 587 ± 38 pA (n = 7, *p* < 0.05) from a control value of 456 ± 27 pA (n = 7). Moreover, as GV-58 was continually present, subsequent addition of riluzole (10 μM) strikingly reduced the magnitude of GV-58-stimulated *I*_Na_ in NSC-34 cells (Figure 12B). Riluzole has been reportedly demonstrated to suppress *I*_Na_ effectively and to be useful in the management of amyotrophic lateral sclerosis or degenerative cervical myelopathy [33,34,35]. These experimental observations reflect that the ameliorating effect of GV-58 on neuromuscular weakness could partly derive from its stimulation of *I*_Na_ demonstrated herein, although the overarching activation of neuronal Ca_V_ channels caused by this compound has been demonstrated [2,3,4,5].

## 4. Discussion

The notable findings in this work are that: (a) GH_3_ cell exposure to GV-58 could differentially increase peak and late components of *I*_Na_ activated by abrupt step depolarization in a concentration-, time-, and state-dependent manner (Figure 1); (b) the *I*_Na_ activated by brief depolarizing pulse was sensitive to either blockage by tetrodotoxin or stimulation by GV-58, but it failed to be affected by ω-conotoxin MVIID (Figure 3) (however, no change in the steady-state *I–V* relation of peak *I*_Na_ in the presence of GV-58 was noted (Figure 2)); (c) the recovery of *I*_Na_ inactivation induced with varying interpulse intervals was enhanced in its presence, while *I*_Na_ decay evoked by a train of depolarizing pulses became slowed (Figure 4); (d) the decline of peak *I*_Na_ during rapid repetitive stimuli was slowed in the presence of GV-58 (Figure 5), while this compound stimulated peak *I*_Na_ in a tonic and use-dependent manner (Figure 6); (e) cell exposure to GV-58 was able to enhance *I*_Na(R)_ and *I*_Na(W)_ evoked by the descending and ascending ramp pulses, respectively (Figure 7 and Figure 8); (f) the presence of this compound decreased *I*_K(A)_ together with an increase in inactivation rate of the current, while it mildly reduced *I*_K(erg)_ measured throughout the entire voltage-clamp steps delivered (Figure 9 and Figure 10); (g) GV-58 increased the frequency of spontaneous APs recorded under current-clamp conditions (Figure 11); and (h) the *I*_Na_ amplitude in NSC-34 motor neuron-like cells could be enhanced by adding GV-58 (Figure 12). The mRNA transcripts for the α-subunit of Na_V_1.1, Na_V_1.2, and Na_V_1.6 were reported to be present in GH_3_ cells [11]. Together, findings from this study can be interpreted to reflect that the presence of GV-58 can stimulate the amplitude and gating of *I*_Na_ and that it would engage in the modifications of spontaneous APs in electrically excitable cells (e.g., GH_3_ or NSC-34 cells), presuming that similar in vivo results occur.

In the current study, we disclosed that the presence of GV-58 was able to increase recovery of *I*_Na_ inactivation evoked in response to varying interpulse intervals in a geometrics-based progression; however, it slowed down current inactivation activated during pulse-train stimulation. Therefore, the post-spike *I*_Na_ during rapid repetitive stimuli is virtually linked to the prominent slow inactivation in GH_3_ cells, suggesting such slow inactivation develops from open channels, and, if recovery from slow inactivation occurs through the open state (conformation), it would be accompanied by a small residual steady current. The presence of GV-58 would be anticipated to enhance the magnitude of post-spike and steady currents, hence raising the occurrence of subthreshold potential [21,24,36,37]. Likewise, the magnitude of ramp-induced *I*_Na(R)_ and *I*_Na(W)_ residing in GH_3_ cells was expected to become elevated during exposure to GV-58. The simulation by GV-58 of firing frequency recorded under current-clamp experiments is consistent with a previous study made on microelectrode arrays [4]. It is thus reasonable to assume that the GV-58 molecule may have a higher affinity to the open/inactivated state than to the resting (closed) state residing in the Na_V_ channels, although the detailed ionic mechanism of its stimulatory actions on this channel warrants further detailed investigations.

GV-58 has been previously shown to be more potent on N- and P/Q-type Ca^2+^ channels with an EC_50_ of 6.8 and 9.9 μM, respectively, over L-type Ca^2+^ channels (EC_50_ > 100 μM) [1]. Additionally, several lines of evidence have been revealed to indicate that GV-58 can stimulate N- and P/Q-type Ca^2+^ currents [2,3,4,5]. Pituitary cells have been demonstrated to functionally express two components of Ca_V_-channel currents found in GH_3_ cells [11,38,39]. As such, the question arises as to whether the stimulatory effect of GV-58 on *I*_Na_ or AP firing observed in GH_3_ cells may actually result from the activation of Ca^2+^ currents. However, this notion appears to be difficult to reconcile with the current observations revealing that ω-conotoxin MVIID, a blocker of N- and P/Q-type Ca_V_-channel currents [20], failed to suppress *I*_Na_ and that further addition of ranolazine, an inhibitor of late *I*_Na_ [16,19], was able to counteract GV-58-stimulated AP firing. It is also important to notice that GV-58 could not only inhibit the amplitude of *I*_K(A)_ but also decrease the inactivation time constant of the current, although it mildly suppressed *I*_K(erg)_. Therefore, under our experimental conditions, the stimulation of *I*_Na_, *I*_Na(R)_, and *I*_Na(W)_ produced by GV-58 tends to occur in a manner largely unlinked to its agonistic actions on Ca_V_-channel currents. The GV-58 molecule can exert an interaction at the binding site(s) residing preferentially on Na_V_ channels. In this scenario, the accentuation of spontaneous APs caused by the GV-58 existence could result primarily from the accentuation of *I*_Na_, including peak or late *I*_Na_, *I*_Na(R)_, and *I*_Na(W)_.

Concentration-dependent stimulation of peak and late *I*_Na_ with effective EC_50_ values of 8.9 and 2.6 μM was, respectively, observed in the presence of GV-58. As cells were exposed to GV-58, the amplitude of peak *I*_Na_ was increased in combination with a substantial increase in the τ_inact(S)_ value of *I*_Na_ activation in response to abrupt membrane depolarization. It is also worth noting, from the present observations, that, since EC_50_ values detected in this study tend to be lower than EC_50_ needed for its activation of N- and P/Q-type Ca_V_ channels [1], the GV-58 actions on *I*_Na_ and *I*_K(A)_ reported herein are thus likely to be therapeutically or pharmacologically relevant. Moreover, in our study, in NSC-34 motor neuron-like cells, the exposure to GV-58 not only augmented peak and late *I*_Na_ but also raised the inactivation time constant of the current. However, whether GV-58-induced amelioration of neuromuscular weakness is pertinent to its stimulatory effect on the amplitude and/or kinetic gating of *I*_Na_ intrinsically in motor neurons needs to be further delineated. Alternatively, to what extent other structurally related compounds of GV-58 (e.g., ML-50 or GV-05) have any propensity to influence the amplitude and gating of *I*_Na_ is also worthy of being explored further.

In view of the experimental observations demonstrated herein, the perturbations by GV-58 of transmembrane ionic currents such as *I*_Na_ and *I*_K(A)_ tend to be important and, thus, lend credence to the notion that those actions might be in part associated with its actions on the behaviors (e.g., spontaneous APs) in electrically excitable cells [15,36]. The loss of function in *I*_Na_ (e.g., Na_V_1.4-encoded current) has been recently demonstrated to be intimately linked to different neuromuscular disorders (e.g., sodium channel weakness or sodium channel myotonia) [40]. Treatment with 3,4-diaminopyridine base was also revealed to be effective in improving muscle weakness of Lambert–Eaton myasthenia [27]. Awareness thus needs to be stressed in attributing its growing use specifically to the selective agonistic effect on N- and P/Q-type Ca_V_ channels, since previous studies appear to underscore the importance of the increase in *I*_Na_ and the decrease in *I*_K(A)_ during the presence of GV-58.

## Figures and Tables

**Table 1:** Composition of normal Tyrode’s solution and the pipette solution used in this study.

**Bathing Solution**	**Purpose or Name**	**Composition**
Bathing solution	Normal Tyrode’s solution	136 mM NaCl, 5.4 mM KCl, 1.8 mM CaCl_2_, 0.53 mM MgCl_2_, 5.5 mM glucose, and 5.5 mM HEPES-NaOH buffer, pH 7.4
Bathing solution	High-K^+^, Ca^2+^-free solution	130 mM KCl, 10 mM NaCl, 3 mM MgCl_2_, 6 mM glucose, 5 mM HEPES-KOH buffer, pH 7.4
Pipette solution	For recordings of K^+^ current or membrane potential	130 mM K-aspartate, 20 mM KCl, 1 mM KH_2_PO_4_, 1 mM MgCl_2_, 3 mM Na_2_ATP, 0.1 mM Na_2_GTP, 0.1 mM EGTA, and 5 mM HEPES-KOH buffer, pH 7.2
Pipette solution	For recordings of Na^+^ or Ca^2+^ current	130 mM Cs-aspartate, 20 mM CsCl, 1 mM KH_2_PO_4_, 1 mM MgCl_2_, 3 mM Na_2_ATP, 0.1 mM Na_2_GTP, 0.1 mM EGTA, and 5 mM HEPES-CsOH buffer, pH 7.2

## Data Availability

The original data are available upon reasonable request to the corresponding author.

## Abbreviations

ANOVA	analysis of variance
AP	action potential
Ca_V_ channel	voltage-gated Ca^2+^ channel
EC_50_	concentration required for 50% stimulation
*erg*	*ether-à-go-go*-related gene
GV-58	(2*R*)-2-[(6-{[(5-methylthiophen-2-yl)methyl]amino}-9-propyl-9H-purin-2-yl)amino]butan-1-ol
*I–V*	current versus voltage
*I*_Ca,L_	L-type Ca^2+^ current
*I*_K(A)_	A-type K^+^ current
*I*_K(erg)_	*erg*-mediated K^+^ current
*I*_Na_	voltage-gated Na^+^ current
*I*_Na(R)_	resurgent *I*_Na_
*I*_Na(W)_	window *I*_Na_
Na_V_ channel	voltage-gated Na^+^ channel
Ran	ranolazine
Ril	riluzole
τ_inact(S)_	slow component in the inactivation rate constant
TTX	tetrodotoxin
